# Use of Multimodal Imaging and Clinical Biomarkers in Presymptomatic Carriers of *C9orf72* Repeat Expansion

**DOI:** 10.1001/jamaneurol.2020.1087

**Published:** 2020-05-18

**Authors:** Joke De Vocht, Jeroen Blommaert, Martijn Devrome, Ahmed Radwan, Donatienne Van Weehaeghe, Maxim De Schaepdryver, Jenny Ceccarini, Ahmadreza Rezaei, Georg Schramm, June van Aalst, Adriano Chiò, Marco Pagani, Daphne Stam, Hilde Van Esch, Nikita Lamaire, Marianne Verhaegen, Nathalie Mertens, Koen Poesen, Leonard H. van den Berg, Michael A. van Es, Rik Vandenberghe, Mathieu Vandenbulcke, Jan Van den Stock, Michel Koole, Patrick Dupont, Koen Van Laere, Philip Van Damme

**Affiliations:** 1 KU Leuven, Department of Neurosciences, Experimental Neurology, B-3000 Leuven, Belgium; 2 KU Leuven, University Hospitals Leuven, University Psychiatric Center, Adult Psychiatry, B-3000 Leuven, Belgium; 3 University Hospitals Leuven, Department of Neurology, B-3000 Leuven, Belgium; 4 VIB - Center of Brain & Disease Research, Laboratory of Neurobiology, B-3000 Leuven, Belgium; 5 KU Leuven, Department of Oncology, B-3000 Leuven, Belgium; 6 KU Leuven, University Hospitals Leuven, Department of Imaging and Pathology, Division of Nuclear Medicine, B-3000 Leuven, Belgium; 7 KU Leuven, Department of Imaging and Pathology, Translational MRI, B-3000 Leuven, Belgium; 8 KU Leuven, Department of Neurosciences, Laboratory for Molecular Neurobiomarker Research, B-3000 Leuven, Belgium; 9 ALS Center, Rita Levi Montalcini Department of Neuroscience, University of Turin, Turin, Italy; 10 Institute of Cognitive Sciences and Technologies, CNR, Rome, Italy; 11 Medical Radiation Physics and Nuclear Medicine, Karolinska University Hospital, Stockholm, Sweden; 12 KU Leuven, Leuven Brain Institute, Laboratory for Translational Neuropsychiatry, B-3000 Leuven, Belgium; 13 KU Leuven, University Psychiatric Center, Geriatric Psychiatry, B-3000 Leuven, Belgium; 14 University Hospitals Leuven, Center for Human Genetics, B-3000 Leuven, Belgium; 15 Brain Center Rudolf Magnus, Department of Neurology, University Medical Center Utrecht, Utrecht, the Netherlands; 16 KU Leuven, Department of Neurosciences, Laboratory for Cognitive Neurology, B-3000 Leuven, Belgium

## Abstract

**Importance:**

During a time with the potential for novel treatment strategies, early detection of disease manifestations at an individual level in presymptomatic carriers of a hexanucleotide repeat expansion in the *C9orf72* gene (preSxC9) is becoming increasingly relevant.

**Objectives:**

To evaluate changes in glucose metabolism before symptom onset of amyotrophic lateral sclerosis or frontotemporal dementia in preSxC9 using simultaneous fluorine 18–labeled fluorodeoxyglucose ([^18^F]FDG positron emission tomographic (PET) and magnetic resonance imaging as well as the mutation’s association with clinical and fluid biomarkers.

**Design, Setting, and Participants:**

A prospective, case-control study enrolled 46 participants from November 30, 2015, until December 11, 2018. The study was conducted at the neuromuscular reference center of the University Hospitals Leuven, Leuven, Belgium.

**Main Outcomes and Measures:**

Neuroimaging data were spatially normalized and analyzed at the voxel level at a height threshold of *P* < .001, cluster-level familywise error–corrected threshold of *P* < .05, and statistical significance was set at *P* < .05 for the volume-of-interest level analysis, using Benjamini-Hochberg correction for multiple correction. W-score maps were computed using the individuals serving as controls as a reference to quantify the degree of [^18^F]FDG PET abnormality. The threshold for abnormality on the W-score maps was designated as an absolute W-score greater than or equal to 1.96. Neurofilament levels and performance on cognitive and neurologic examinations were determined. All hypothesis tests were 1-sided.

**Results:**

Of the 42 included participants, there were 17 with the preSxC9 mutation (12 women [71%]; mean [SD] age, 51 [9] years) and 25 healthy controls (12 women [48%]; mean [SD] age, 47 [10] years). Compared with control participants, significant clusters of relative hypometabolism were found in frontotemporal regions, basal ganglia, and thalami of preSxC9 participants and relative hypermetabolism in the peri-Rolandic region, superior frontal gyrus, and precuneus cortex. W-score frequency maps revealed reduced glucose metabolism with local maxima in the insular cortices, central opercular cortex, and thalami in up to 82% of preSxC9 participants and increased glucose metabolism in the precentral gyrus and precuneus cortex in up to 71% of preSxC9 participants. Other findings in the preSxC9 group were upper motor neuron involvement in 10 participants (59%), cognitive abnormalities in 5 participants (29%), and elevated neurofilament levels in 3 of 16 individuals (19%) who underwent lumbar puncture.

**Conclusions and Relevance:**

The results suggest that [^18^F]FDG PET can identify glucose metabolic changes in preSxC9 at an individual level, preceding significantly elevated neurofilament levels and onset of symptoms.

## Key Points

**Question:**

Can metabolic brain changes be detected in presymptomatic individuals who are carriers of a hexanucleotide repeat expansion in the *C9orf72* gene (preSxC9) using time-of-flight fluorine 18–labeled fluorodeoxyglucose positron emission tomographic imaging and magnetic resonance imaging, and what is the association between the mutation and clinical and fluid biomarkers of amyotrophic lateral sclerosis and frontotemporal dementia?

**Findings:**

In a case-control study including 17 preSxC9 participants and 25 healthy controls, fluorine 18–labeled fluorodeoxyglucose positron emission tomographic imaging noted significant clusters of relative hypometabolism in frontotemporal regions, the insular cortices, basal ganglia, and thalami in the preSxC9 participants. Use of this strategy allowed detection of changes at an individual level.

**Meaning:**

Glucose metabolic changes appear to occur early in the sequence of events leading to manifest amyotrophic lateral sclerosis and frontotemporal dementia. Fluorine 18–labeled fluorodeoxyglucose positron emission tomographic imaging may provide a sensitive biomarker of a presymptomatic phase of disease.

## Teaser

This case-control study examines the use of fluorine 18–labeled fluorodeoxyglucose positron emission tomographic imaging to detect glucose metabolic changes in individuals who are presymptomatic carriers of a mutation in the *C9orf72* gene that leads to amyotrophic lateral sclerosis and frontotemporal dementia.

## Introduction

Amyotrophic lateral sclerosis (ALS) and frontotemporal dementia (FTD) are related neurodegenerative disorders. Amyotrophic lateral sclerosis primarily affects the motor system with upper and lower motor neuron involvement, but extramotor manifestations may occur.^1,2,3^ Frontotemporal dementia is the second most common form of presenile dementia, caused by degeneration of frontal and anterior temporal cortices. It affects brain regions implicated in executive control, language, behavior, and personality.^4^ The disease course of both ALS and FTD is progressive and invariably fatal. The molecular link between ALS and FTD has been confirmed by the discovery of the hexanucleotide repeat expansions in the 3′ untranslated region of the chromosome 9 open reading frame 72 gene (*C9orf72*, OMIM 614260), the most common known monogenetic cause of both ALS and FTD.^5,6,7^

During this time of antisense oligonucleotides and other interventional gene therapies, research in the presymptomatic stage may contribute to the development of novel treatment strategies^8^ and detection of individuals at risk of developing ALS and/or FTD, and ultimately lay the foundation for future clinical studies to slow or even prevent clinical disease manifestation.^9^ Presymptomatic carriers of disease-causing mutations permit in vivo research of the brain at a unique time to gain a better understanding of the early mechanisms that precede the onset of symptoms.

Over the past 10 years, study findings have suggested that several neurodegenerative diseases are preceded by an intermediate presymptomatic phase.^10,11^ Research in presymptomatic carriers of a hexanucleotide repeat expansion in the *C9orf72* gene (preSxC9) reported the occurrence of cognitive and behavioral changes, neuropsychiatric symptoms, and degeneration of gray matter (GM) and white matter (WM).^12,13,14,15,16,17,18,19,20,21^

Neurofilaments (Nfs), such as neurofilament light chain (NfL) and phosphorylated neurofilament heavy chain (pNfH), have been studied extensively in ALS and FTD. Elevated levels of NfL and pNfH, both markers of neuronal injury and neurodegeneration, demonstrated high diagnostic performance.^22^ Previous research has shown that NfL is increased in symptomatic, but not presymptomatic, preSxC9 at the group level.^23^ Recent studies suggested that a slow increase in Nf levels can be observed in presymptomatic individuals who carry the mutation as far as 3.5 years before diagnosable illness,^24,25,26^ while another study described an association between higher NfL levels and GM atrophy.^27^

It has often been suggested that assessing glucose metabolism using positron emission tomographic (PET) imaging with fluorine 18–labeled fluorodeoxyglucose ([^18^F]FDG) is a useful diagnostic marker in the earliest stage of ALS and FTD.^28,29,30,31^ Moreover, [^18^F]FDG PET imaging serves as a relevant biomarker for disease staging, cognitive impairment, and survival prediction.^29,32^

However, little is known about the glucose metabolic changes that may occur before clinical disease manifestation in preSxC9. The goal of our study was to evaluate changes in glucose metabolism that occur before diagnosable illness, ie, the presymptomatic disease stage,^33^ in preSxC9. In addition, we wanted to explore the association between cerebral glucose metabolism and other known indicators of disease, such as Nf levels in cerebrospinal fluid (CSF), neuropsychological capacities, and clinical neurologic examination.

## Methods

### Participants

A total of 29 healthy individuals serving as controls were included in this study, of whom 25 were considered in the analysis. None of the volunteers had a first-degree relative with dementia or a history of neurologic illness, psychiatric illness, or substance use. Participants with brain lesions noted on structural magnetic resonance imaging (MRI) were excluded. Demographic characteristics are detailed in Table 1.

**Table 1.** Demographics and Clinical Data of PreSxC9 and Control Group

Characteristic	PreSxC9 (n=17)	Healthy controls (n=25)	Statistical test for group difference	*P* value
Age, mean (SD), y	51 (9)	47 (10)	Mann-Whitney, 154	.13
Sex, No. (%)				
Women	12 (71)	12 (48)	χ^2^_1_ = 2.11	.13
Men	5 (29)	13 (52)		
Educational level, ISCED, No. (%)^a^				
0-4	7 (41)	8 (32)	χ^2^_1_ = 0.37	.39
5-6	10 (59)	17 (68)	NA	NA
MMSE score, median (range)^b^	29 (26-30)	30 (28-30)	Mann-Whitney, 156.5	.12
BDI, median (range)^c^	NA	2 (0-5)	NA	NA
Psychiatric drugs, No. (%)	1 (6)	NA	NA	NA
Antidepressants, No. (%)	1 (6)	NA	NA	NA

Abbreviations: BDI, Beck Depression Inventory; ISCED, International Standard Classification of Education Scale; MMSE, Mini-Mental-State Examination; NA, not applicable; preSxC9, presymptomatic carrier of a hexanucleotide repeat expansion in the *C9orf72* gene.

^a^ Categorized according to the ISCED 1997 definitions. Scale numbers represent nontertiary education, 0-4, and tertiary education, 5-6.

^b^ Total score ranges, 0 to 30; lower scores indicate worse cognitive function.

^c^ Total score ranges, 0 to 63; higher scores indicate more severe depressive symptoms.

The study was conducted from November 30, 2015, to December 11, 2018, at the neuromuscular reference center of the University Hospitals Leuven, Leuven, Belgium. All participants provided written informed consent, and this study was approved by the ethics committee of the University Hospitals Leuven, Leuven, Belgium. This study followed the Strengthening the Reporting of Observational Studies in Epidemiology (STROBE) reporting guideline for case-control studies.

We compared the Nf levels in the preSxC9 group with those of a control group (n = 10; mean [SD] age, 49 [14] years) previously reported.^34^ A consecutive series of 17 preSxC9 participants was included in this study. A pathogenic expansion of *C9orf72* was considered as having more than 30 repeats. All preSxC9 participants were native Flemish speakers, and their educational levels were between 3 ([upper] secondary education) and 6 (second stage of tertiary education) on the International Standard Classification of Education scale.^35^ None of the preSxC9 participants met the clinical diagnostic criteria for ALS or FTD.^36,37^ Exclusion criteria were the presence of clinically apparent ALS or FTD, severe and chronic illness, substance use, and traumatic brain injury.

All participants with preSxC9 were evaluated with the Dutch version of the Edinburgh Cognitive and Behavioral ALS Screen (ECAS) by an experienced neuropsychologist (J.D.V.).^35^ The ECAS is a brief, multidomain screening battery that assesses cognitive functions typically affected in patients with ALS (language, verbal fluency, and executive functioning), as well as cognitive functions not typically affected in patients with ALS (memory, visuospatial functioning).^38^ Dutch normative data were used, with the fifth percentile as a threshold for abnormality.^35^ Results of the ECAS are presented in Table 2.

**Table 2.** Clinical Characteristics in PreSxC9 Participants

Characteristic	Median (range)	Percentile rank scores ≤5%, No. (%)
ECAS performance^a^		
Total score	117 (93-124)	1 (6)
ALS specific^b^		
Total score	86 (69-92)	1 (6)
Language	28 (25-28)	0
Verbal fluency	20 (14-20)	1 (6)
Executive functions	39 (26-44)	3 (18)
ALS nonspecific^c^		
Total score (of 36)	30 (23-33)	0
Memory (of 24)	18 (11-21)	1 (6)
Visuospatial functions (of 12)	12 (11-12)	0

Abbreviations: ALS, amyotrophic lateral sclerosis; ECAS, Edinburgh Cognitive and Behavioral ALS Screen; preSxC9, presymptomatic carrier of a hexanucleotide repeat expansion in the *C9orf72* gene.

^a^ Possible score range, 0 to 136; lower scores indicate more severe cognitive dysfunction.

^b^ Evaluates functions typically affected in ALS. Total score ranges from 0 to 100; language, 0 to 28; verbal fluency, 0 to 20; and executive functions, 0 to 42. Lower scores indicate more severe cognitive dysfunction.

^c^ Evaluates cognitive functions not typically affected in ALS. Total score ranges from 0 to 36; memory, 0 to 24; and visuospatial functions, 0 to 12. Lower scores indicate more severe cognitive dysfunction.

All of preSxC9 participants underwent a standard clinical neurologic examination by a neurologist experienced in neuromuscular disorders (P.V.D.).

Sixteen of 17 preSxC9 participants agreed to undergo a lumbar puncture according to standardized protocol at the University Hospitals Leuven to determine the Nf levels in the CSF within 48 hours following the [^18^F]FDG PET MRI scan. Neurofilament levels in CSF were measured using commercially available kits for NfL (UD51001, with an intraassay variability of 1.6% and interassay variability of 8.7%; UmanDiagnostics AB) and pNfH (with an intraassay variability of 5.2% and interassay variability of 8.7%; Euroimmun AG). Assessment of Nf levels was done using predefined diagnostic cutoff values for NfL (1227 pg/mL)^34^ and pNfH (750 pg/mL).^39^

All participants underwent simultaneous [^18^F]FDG PET and MR imaging on a hybrid scanner (Signa PET/MR, release MP26; GE Healthcare) with integrated time-of-flight PET scan acquisition and 3-T MRI. All participants fasted for at least 6 hours before [^18^F]FDG administration. [^18^F]FDG was injected intravenously as a bolus (mean [SD], controls: 153 [11] MBq; preSxC9: 149 [6] MBq).

The [^18^F]FDG PET images were acquired in list mode for 25 minutes (30 minutes postinjection). The PET images were reconstructed with ordered subset maximum likelihood expectation maximization with 4 iterations and 28 subsets followed by postfiltering with 4.5-mm gaussian postsmoothing in the transaxial direction and standard smoothing along the Z direction. Images had an initial voxel size of 1.56 × 1.56 × 2.78 mm^3^. A vendor-provided, atlas-based method was used for attenuation correction.^40^

Simultaneous to the PET acquisition, a 3-dimensional volumetric sagittal T1-weighted image (3D BRAVO, repetition time/echo time [TR/TE] = 8.5/3.2 milliseconds, 0.6 × 1 × 1 mm^3^ voxel size, dimensions: 312 × 256 × 256 voxels) and T2-weighted fluid-attenuated inversion recovery image (3D CUBE, TR/TE = 8500/130 milliseconds, 0.7 × 1 × 1 mm^3^ voxel size, dimensions: 268 × 256 × 256 voxels) were acquired.

### Statistical Analysis 

Statistical analyses of clinical data were performed using SPSS software, version 25 (IBM Software) and GraphPad Prism, version 8.0 (GraphPad Software). Demographic characteristics and clinical test results were compared between groups using a χ^2^ test for dichotomous and categorical variables or Mann-Whitney test for numeric variables. All hypothesis tests were 1-sided, and statistical significance was set at *P* < .05.

### Image Analysis

ANTS, version 2.1.0., and SPM, version 12 (Wellcome Trust Centre for Neuroimaging) software, combined with in-house scripts implemented in Matlab (R2018b; The MathWorks Inc), were used to process the T1-weighted and fluid-attenuated inversion recovery images. After visual inspection of the raw T1 images, the T1 images were processed in native space using the antsCorticalThickness pipeline in ANTS,^39^ which performs a brain extraction and segments the image of the individual’s brain by means of 5 specific tissue priors: CSF, cortical GM, WM, subcortical GM, and the brainstem. After visual inspection of the segmentations, 3 control scans were excluded because of poor image quality and subsequent suboptimal brain data extraction and segmentation. Gray matter tissue probability maps were warped to the Nathan Kline Institute template (Rockland Sample, dimensions = 182 × 218 × 182 voxels), which was warped to Montreal Neurological Institute space (voxel size = 1 × 1 × 1 mm^3^, matrix = 182 × 218 × 182) and modulated with the jacobian warp parameters, all using nonlinear symmetric diffeomorphic registration.

All [^18^F]FDG PET images were first quality checked for complete acquisition and motion, then dynamically reconstructed and corrected for potential head motion. The frames, which were reconstructed over a series of 5 minutes, were then averaged. After visual inspection, PET images were coregistered to their respective native MRI and spatially normalized to Montreal Neurological Institute space using ANTS, applying the normalization parameters described above. After visual inspection, 1 control scan was considered an outlier (>3 SD from the mean) and subsequently excluded. The [^18^F]FDG PET images were corrected for partial volume effects with the Müller-Gartner method (PMOD, version 3.9), which considers both GM spill-out and WM spill-in based on the MRI-based GM and WM tissue probability maps. Partial volume correction (PVC) was done using a point-spread function with a 5.5-mm isotropic full width at half maximum to mimic the PET image resolution, while a regression approach was applied for all voxels with a WM probability greater than 0.95 to determine the [^18^F]FDG uptake in WM. The GM probability threshold was set at 0.3 to correct uptake values of GM voxels for WM activity. Partial volume effect–corrected [^18^F]FDG PET images were spatially normalized to Montreal Neurological Institute space using ANTS, applying the normalization parameters described above.

For the group comparison, both MR and [^18^F]FDG PET images were smoothed with an isotropic gaussian smoothing kernel of 8-mm full width at half maximum to blur individual variations. Owing to a difference in ambient conditions (ie, visual input) following tracer injection, the occipital lobe was excluded from all neuroimaging analyses. All PET images were proportionally normalized to the average activity in a GM mask generated from the voxel-based morphometry comparative analysis applying an absolute threshold of 0.1 (excluding the occipital lobe).

The spatially normalized and smoothed images were then entered into a generalized linear model. All hypothesis tests were 1-sided, with a height threshold of *P* < .001 and a cluster-level familywise error (FWE)-corrected threshold of *P* < .05, applying a minimum extent threshold of 150 voxels for the [^18^F]FDG PET analyses. Age was included as a nuisance covariate in [^18^F]FDG PET analyses as well as in the voxel-based morphometry analyses, where total intracranial volume was also considered a nuisance variable. The Talairach atlas^41^ was used to define Brodmann areas and the Harvard-Oxford Atlas^42,43,44,45^ was used for the anatomic localization of significant clusters.

A volume-of-interest–based analysis after region-based voxelwise correction for GM atrophy was conducted using the Hammers N30R83 maximum probability atlas to confirm our findings at the voxel level in PMOD, version 3.9 (PMOD Inc) and SPSS, version 25 (IBM Software). We applied the Benjamini-Hochberg method to correct for multiple testing.

### W-Score Maps

W-score maps ([raw value for each patient − value expected in the control group for the patient’s age] / SD of the residuals in the control group) were computed for preSxC9 using the control group as a reference to quantify the degree of [^18^F]FDG PET imaging abnormality at the voxel level. W-score maps are analogous to *z*-score maps, adjusted for covariates of interest. For our study, we considered age as a covariate of interest.^46^ The threshold for abnormality was defined as an absolute W-score greater than or equal to 1.96, which corresponds to 95% of the area under the curve in a normal distribution. Hypometabolic maps, binarized at a W-score less than or equal to −1.96, and hypermetabolic maps, binarized at a W-score greater than or equal to 1.96, were summed across participants to generate W-score frequency maps to illustrate the fraction of preSxC9 participants surpassing the threshold for abnormality at the voxel level.

## Results

A total of 46 participants (17 preSxC9 and 29 healthy controls) were included in this study. After data inspection, all preSxC9 participants (mean [SD] age, 51 [9] years; 12 women [71%], 5 men [29%]) and 25 (4 of 29 excluded owing to poor image quality) healthy controls (mean [SD] age, 47 [10] years; 12 women [48%], 13 men [25%]) were considered for the analyses. The demographics of the study population are given in Table 1. The preSxC9 and control groups did not differ significantly in sex distribution (χ^2^_1_ = 2.11; *P* = .13), educational level (χ^2^_1_ = 0.37; *P* = .39), or age (Mann-Whitney, 1.54; *P* = .13).

### Neuroimaging 

**Figure 1.**
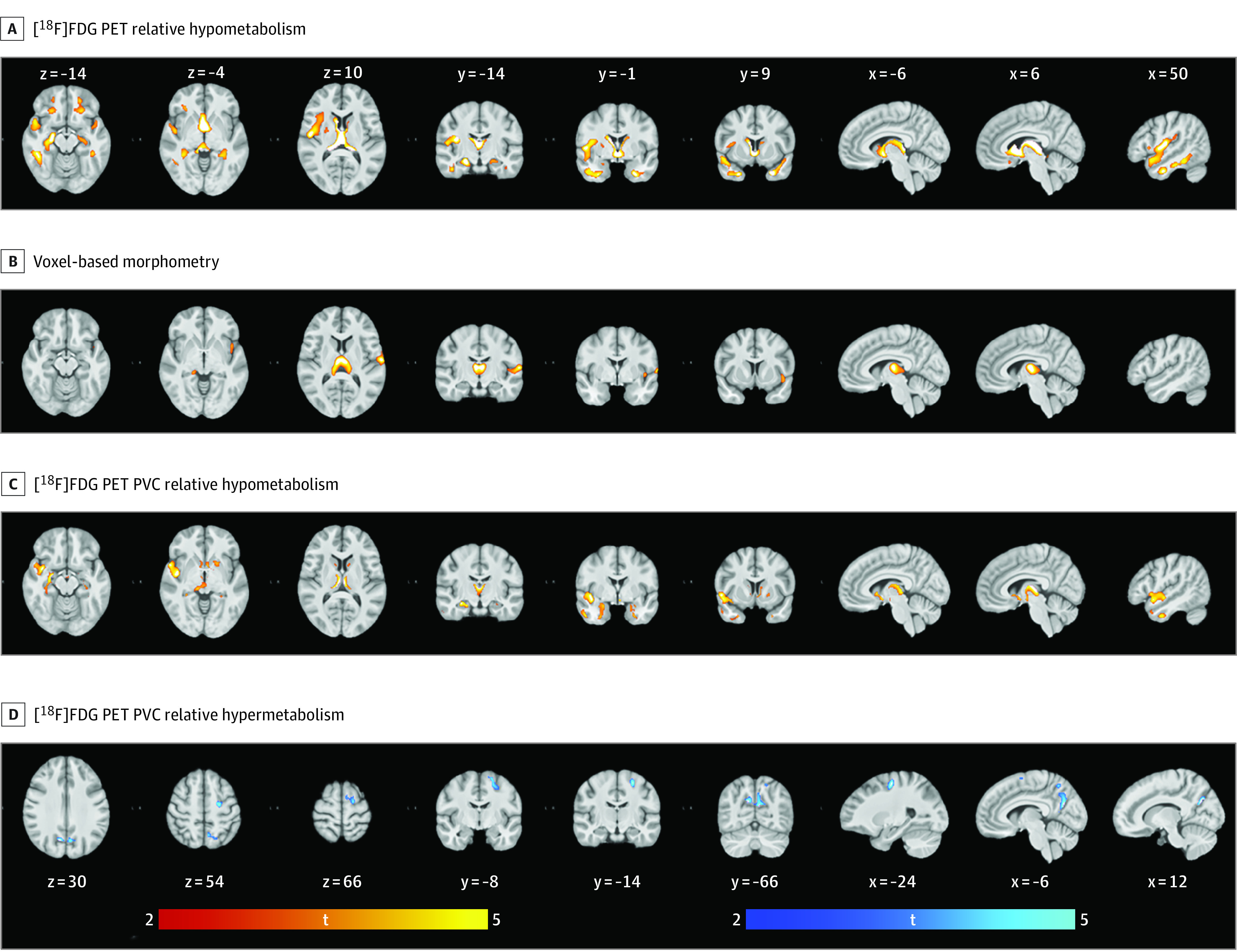
Relative Glucose Metabolism and Gray Matter Volume in Presymptomatic Carriers of a Hexanucleotide Repeat Expansion in the *C9orf72* Gene (PreSxC9) Corrected for Age on Axial, Coronal, and Sagittal Sections Reduced glucose metabolism and gray matter volume depicted in red-yellow, and increased glucose metabolism depicted in blue-white. Data were analyzed at a height threshold of *P* < .001 and were cluster level corrected for familywise error at *P* < .05. A, Projections of areas with relative hypometabolism in preSxC9 participants vs healthy controls. B, Volume decline in preSxC9 participants vs healthy controls. C, Relative hypometabolism in preSxC9 participants and healthy controls following voxel-based PVC. D, Relative hypermetabolism in preSxC9 and healthy controls following voxel-based PVC. [^18^F]FDG indicates fluorine 18–labeled fluorodeoxyglucose; PET, positron emission tomography; PVC, partial volume correction; and t, *t* value. Section numbers refer to Montreal Neurological Institute coordinates.

Relative glucose metabolism was compared between the preSxC9 and control cohorts. This analysis revealed significant clusters of relative hypometabolism in the preSxC9 group compared with the control group (range, 27%-36%) situated in the basal ganglia, thalamus, and frontotemporal and insular cortices. All analyses were thresholded at a height of *P* < .001 and FWE-corrected level of *P* < .05 at the cluster level (Figure 1; eTable 1 in the Supplement). At the group level, we observed no significant clusters of relative hypermetabolism.

The comparative voxel-based volumetric analysis (voxel-based morphometry) revealed significant clusters of reduced GM volume (range, 19%-25%) located in the frontotemporal regions, including the peri-Rolandic region, insular cortices, basal ganglia, and thalami. All analyses were thresholded at a height of *P* < .001 and FWE-corrected level of *P* < .05 at the cluster level (Figure 1; eTable 2 in the Supplement). A voxel-based regression analysis of the association between age and GM volume failed to show a significant difference in the slopes of the preSxC9 and control participants.

The [^18^F]FDG PET imaging data were also analyzed with partial volume effect correction to account for GM atrophy. Significant clusters of relative hypometabolism (range, 16%-22%) persisted in frontotemporal regions, including the insular cortices, as well as the basal ganglia and thalami. All analyses were thresholded at a height of *P* < .001 and FWE-corrected level of *P* < .05 at the cluster level (Figure 1; eTable 3 in the Supplement; Video). A voxel-based regression analysis of the association between age and cerebral metabolism failed to show a significant difference in the slopes of the preSxC9 and healthy control participants.

**Video.** Relative Glucose Metabolism on Fluorine 18–Labeled Fluorodeoxyglucose Positron Emission Tomographic (PET) Imaging in PreSxC9 Participants vs Controls Relative hypometabolism and relative hypermetabolism following partial volume correction (PVC) in presymptomatic carriers of a hexanucleotide repeat expansion in the *C9orf72* gene (preSxC9) in relation to healthy controls in red-yellow and blue-white, respectively, while correcting for age (height-corrected threshold of *P* < .001 at a cluster-level familywise error–corrected threshold of *P* < .05).

These findings were supported in a volume-of-interest–based analysis applying region-based voxelwise correction for GM atrophy (eFigure 1 and eTable 5 in the Supplement). Significant clusters of relative hypermetabolism (range, 6%-7%) emerged in the peri-Rolandic region, the superior frontal gyrus, and the precuneus cortex following PVC. All analyses were thresholded at a height of *P* < .001 and FWE-corrected level of *P* < .05 at the cluster level (Figure 1; eTable 4 in the Supplement; Video). To confirm the presence of relative hypermetabolic clusters in preSxC9 participants, the analysis was repeated using standardized uptake value ratio images; cortical uptake was scaled to the average uptake in cerebellar structures not reported as being affected by a mutation in the *C9orf72* gene, supporting our findings (eResults, eFigure 4, eTable 6 in the Supplement).^47^

### Clinical Parameters

Neurologic examination revealed mild signs of upper motor neuron (UMN) abnormalities in 12 of the 17 preSxC9 participants (71%). As the presence of a Hoffman sign or ankle clonus is not necessarily abnormal in young people, we only considered the presence of a jaw jerk, a Babinski sign, hyperreflexia, and increased muscle tone for further analyses. This was apparent overall in 10 preSxC9 participants (59%). In 5 participants (29%) increased muscle tone was observed in the lower extremities; 1 (6%) also presented with increased muscle tone in upper extremities.

Five participants (29%) of the preSxC9 cohort presented with abnormal neuropsychological performance. Executive functioning was affected in 3 preSxC9 participants (18%), 1 participant presented with isolated abnormal performance on verbal fluency, and another individual showed isolated impairment on the memory subdomain (Table 2).

**Figure 2.**
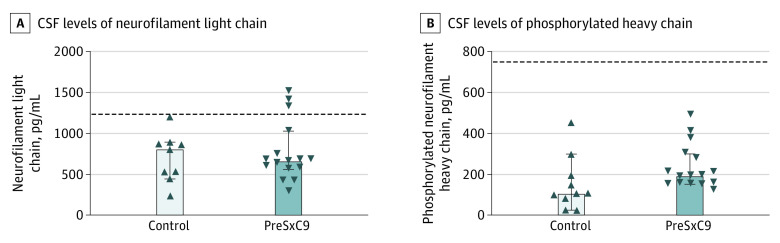
Neurofilament Levels in the Cerebrospinal Fluid (CSF) of Presymptomatic Carriers of a Hexanucleotide Repeat Expansion in the *C9orf72* Gene (PreSxC9) A, Neurofilament light chain in the CSF of healthy controls and preSxC9 participants. B, Phosphorylated neurofilament heavy chain in the CSF of healthy controls and preSxC9 participants. The dotted line indicates diagnostic cutoff level. The upward arrowheads indicate the controls, and the downward arrowheads indicate the preSxC9 participants. Error bars indicate 95% CIs.

Examination of the CSF showed median NfL levels of 652 pg/mL (range, 276-1510) and pNfH levels of 195 pg/mL (range, 123-490). The NfL and pNfH levels did not differ significantly between the preSxC9 and healthy controls at the group level. However, elevated NfL levels, ie, surpassing the diagnostic cutoff, were observed at the individual level in the CSF of 19% of the preSxC9 group (Figure 2). All 3 of the 16 who underwent lumbar puncture displayed signs of UMN involvement on clinical neurologic examination, and 1 of the 3 individuals with elevated Nf levels displayed an abnormal score on the memory domain using the ECAS. The pNfH levels in the CSF remained within the reference range in all preSxC9 participants (Figure 2).

We were unable to identify a significant association between relative tracer uptake and age, UMN involvement, ECAS performance, or Nf levels in CSF at the group level in preSxC9 participants using regression analyses at a height-corrected threshold of *P* < .001 and with a cluster-level FWE-corrected threshold of *P* < .05. Similarly, no significant association was identified between GM volume and age, UMN involvement, ECAS performance, or Nf levels in CSF, applying the same threshold for significance.

**Figure 3.**
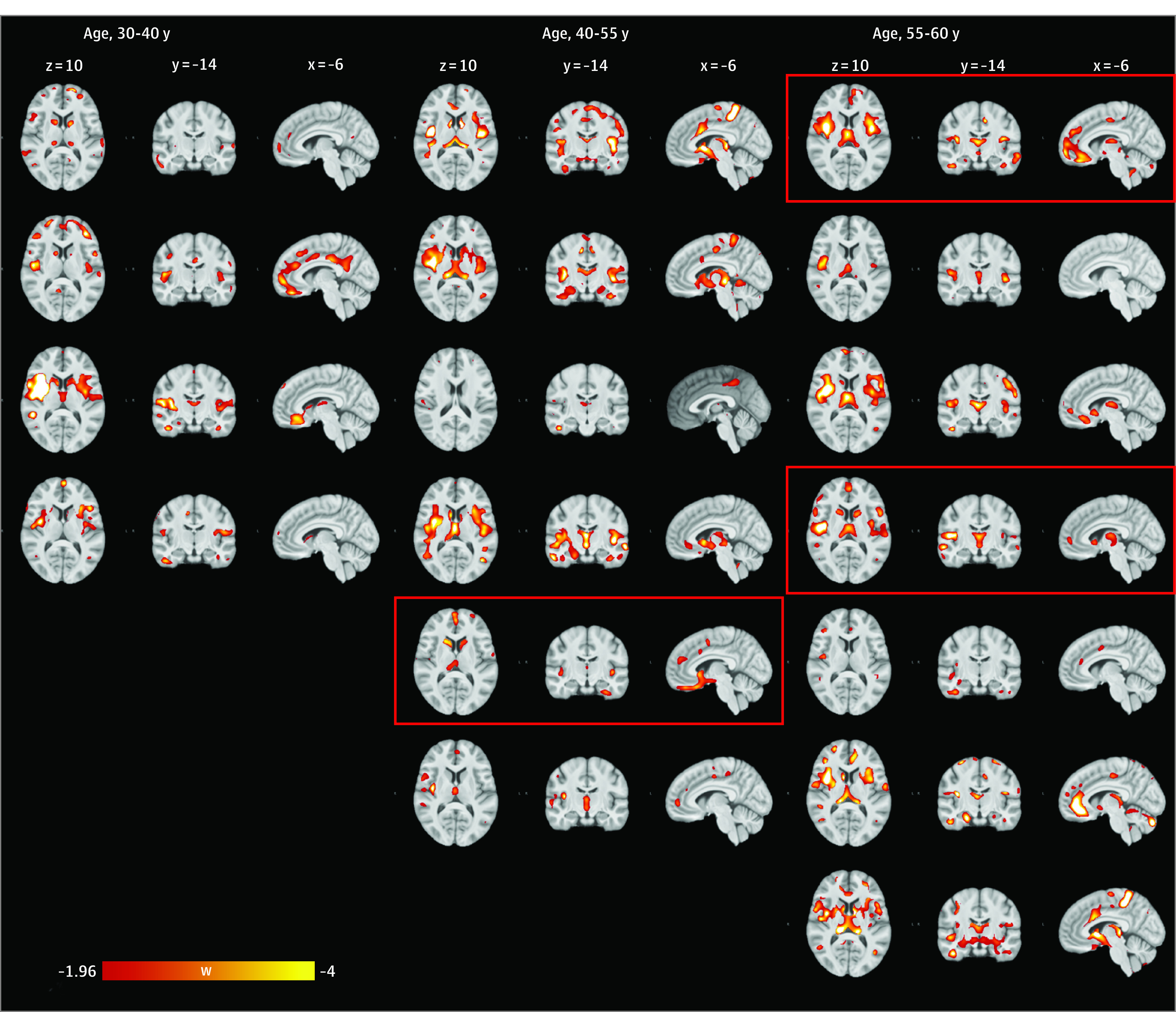
Individual W-Score Maps of Relative Hypometabolism Observed in PreSxC9 Participants Thresholds for W-score maps were set at a W score less than or equal to −1.96 and sorted by age, displayed on axial (z = 10), coronal (y = −14), and sagittal (x = −6) sections. Section numbers refer to Montreal Neurological Institute coordinates. Images from participants with elevated neurofilament levels are within red boxes. w indicates W score.

We generated voxel-level W-score maps to evaluate how many preSxC9 participants presented with suprathreshold voxels in key regions. A frequency image of the W-score maps, generated from the [^18^F]FDG PET images without correcting for partial volume effect, showed that 14 preSxC9 participants (82%) had significantly reduced tracer uptake in the insular cortices, central opercular cortex, and thalami (eFigure 2A in the Supplement). In addition, a frequency image of the W-score maps, generated from the [^18^F]FDG PET images corrected for partial volume effect, showed that up to 71% of preSxC9 patients had significantly increased tracer uptake, surpassing the predefined threshold of an absolute W-score of 1.96, which corresponds to the 2.5th percentile on both sides in the peri-Rolandic region (eFigure 2B in the Supplement). A mean image of the W-score maps in the preSxC9 cohort reflected the consistency of the changes observed at the group level (eFigure 3 in the Supplement). Individual W-score maps of relative hypometabolism supported the pattern observed at the group level in up to 82% of preSxC9 participants (Figure 3). A W-score frequency map of GM volume reduction revealed suprathreshold voxels in the thalami and central opercular cortex in 11 preSxC9 participants (65%) (eFigure 2C in the Supplement). In addition, using the W-score maps, we were unable to identify a clear association between the extent of abnormality and UMN involvement, ECAS performance, and Nf levels in CSF.

## Discussion

A voxelwise comparison of glucose metabolic patterns revealed clusters of relative glucose hypometabolism situated in frontotemporal and insular cortices, the basal ganglia, and thalami. Moreover, GM volume reductions revealed a widespread neuroanatomic signature in the frontotemporal and insular cortices, basal ganglia, and thalami. The observed volumetric differences are consistent with structural changes reported in previous studies of preSxC9.^13^ Even though regional hypometabolism in subcortical and extramotor regions may be explained in part by neuronal loss, the functional disruption identified by [^18^F]FDG PET imaging was supported, as clusters of reduced glucose metabolism in aforementioned regions withstood PVC, and thus correction for GM atrophy.

Significant clusters of relative hypermetabolism were observed in the precentral and superior frontal gyrus and the precuneus cortex following PVC. This finding may be interpreted as compensatory neuronal activity or a possible abnormal function of cortico-striatal-thalamic-cortical circuits resulting in UMN abnormalities. In addition, we can speculate that the observed clusters of relative hypermetabolism reflect neuroinflammation associated with activated astrocytes or microglia.^29^

The observed structural and metabolic changes in the preSxC9 participants suggest that brain regions corresponding to cognitive and motor processes are impaired in the presymptomatic stage of ALS and FTD. These findings are in line with previous [^18^F]FDG PET imaging studies in symptomatic carriers of a *C9orf72* hexanucleotide repeat expansion, demonstrating relative hypometabolism in frontotemporal and subcortical regions.^48,49^ Moreover, our findings support the role of the thalamus in *C9-*related disease.^49,50^

The role of the cerebellum in *C9*-related disease remains unclear. A recent voxel-based morphometry study from the multicenter Genetic Frontotemporal Dementia Initiative consortium described GM volume reductions in the superior-posterior cerebellum.^14^ We, however, did not observe significant GM volume reductions in the cerebellum, supporting the findings of another study.^15^ To our knowledge, there are no consistent findings on volumetric changes in the cerebellum of preSxC9 individuals.

For this study, W-score maps were generated to observe individual effects, as individual differences may have been washed out in a group-level voxel-based analysis. W-score frequency maps reflected the consistency of the pattern observed at the group level in individual W-score maps of a number of preSxC9 participants. These maps demonstrated that the highest frequencies (up to 82%) of reduced glucose metabolic uptake, below the threshold for abnormality, were found in the insular cortices, central opercular cortex, and thalami of preSxC9 participants. The highest frequencies (up to 71%) of increased glucose metabolism, above the threshold for abnormality, following PVC were found in the peri-Rolandic region and superior frontal gyrus of the preSxC9 participants. Given that only part of the preSxC9 cohort had cognitive, pyramidal, or Nf changes, we suggest that the metabolic changes may occur early in the sequence of events leading to manifest ALS and FTD.

Because the age at disease onset is variable in *C9orf72* repeat expansion carriers, the preSxC9 cohort in the present study most likely consists of a mixture of individuals who are relatively close to or far from disease onset. In addition, a hexanucleotide repeat expansion is known to be associated with a clinically heterogeneous disease spectrum.^2^ The conceivable high degree of clinical variability within the preSxC9 group could potentially blur correlations with clinical parameters. As we did not observe an association with deviation from the norm and increasing age, longitudinal studies are needed to establish how the patterns of hypometabolism evolve and their predictive value for clinical disease onset.

We did not identify significant differences in CSF Nf levels at the group level between healthy controls and preSxC9 participants. Other studies were also unable to identify significant differences for this marker for axonal injury between healthy controls and individuals who are preSxC9.^24,51^ At the individual level, NfL appears to be more sensitive than pNfH in the phase preceding diagnosable illness: no preSxC9 participant displayed a pathologic increase in pNfH, but 3 preSxC9 participants displayed abnormally high NfL levels.

We did not identify a significant association between Nf levels, ECAS performance, clinical neurologic screening, and findings on neuroimaging. This finding may, at least in part, be explained by sample size, as few preSxC9 participants presented with cognitive changes, elevated Nf levels, and UMN signs. Changes in cerebral metabolism may also precede clinical signs, which is in line with a recent study describing functional reorganization and network resilience in individuals who are preSxC9.^18^

Longitudinal, multimodal PET and MRI studies are needed to gain a better understanding of the sequence of events that precede diagnosable illness. In addition, no significant association was observed between GM volume and increasing age in the preSxC9 cohort. We could therefore speculate that the observed volumetric differences between the preSxC9 and healthy control participants may represent not only GM atrophy but may, at least in part, indicate neurodevelopmental differences. Adolescent neuroimaging studies could assist in gaining more insight into the natural history of brain development in preSxC9.

### Limitations

This study has limitations. First, the sample size was relatively small, which may have been a factor in the power of group comparisons for signs of upper motor neuron involvement and the association between neuroimaging data and clinical indicators of disease. Second, the absence of convertors in our cohort prevented us from exploring the predictive values of these markers for diagnosis. Third, we did not perform neurologic examinations in the control cohort. We also did not use cognitive screening with the ECAS in the healthy controls; however, we administered a Mini-Mental-State Examination in all participants, which did not reflect any cognitive abnormalities. Fourth, the difference in ambient conditions (visual input) between the preSxC9 and control cohorts necessitated masking the occipital lobe from our comparative analyses between preSxC9 and healthy controls, therefore preventing us from performing a whole-brain, voxel-based comparative analysis. However, to ensure the robustness of the patterns of relative hypometabolism, we performed a second whole-brain analysis in the preSxC9 group. This second analysis revealed the same clusters that we observed previously as well as a cluster of relative hypermetabolism in the occipital lobe, supporting our findings.

## Conclusions

This study showed regional glucose metabolic alterations in presymptomatic carriers of a *C9orf72* hexanucleotide repeat expansion before diagnosable illness that remained after correction for volume differences. Within the preSxC9 cohort on W-score maps of [^18^F]FDG PET images, up to 82% (n = 14) presented with voxels surpassing the threshold of abnormality in key regions, Nf levels were elevated in only 19% (n = 3), deviation from the norm according to ECAS performance was observed in 29% (n = 5), 59% (n = 10) presented with subtle UMN signs, and abnormalities were noted on W-score maps on MR images in 65% (n = 11). The individual W-score image suggests that [^18^F]FDG PET might be able to detect neuronal injury in an earlier stage than motor or cognitive changes or Nf levels.

To our knowledge, this is the first study that closely examines cerebral glucose metabolism in preSxC9 carriers and its association with GM volume and indicators of disease. Our findings suggest that [^18^F]FDG PET imaging could provide a sensitive biomarker of a presymptomatic phase of disease, which can be of relevance for future therapeutic strategies. Multimodal and longitudinal imaging studies with an augmented sample size are needed to gain more insight into the sequence of events in the presymptomatic stage of *C9orf72*-related disease.

## Notes

Supplement. eResults. Supplementary Analysis eReferences eFigure 1. Violin Plots of 18F FDG Uptake in Volumes of Interest, After Correcting for GM Atrophy With PVC, in Healthy Controls and PreSxC9 eFigure 2. W-Score Frequency Maps Detailing the Fraction of PreSxC9 With Suprathreshold W-Scores at Voxel Level eFigure 3. Mean W-Score Images Detailing Tracer Uptake in PreSxC9, Thresholded From 0.5 Standard Deviations From the Norm eFigure 4. Relative Hypermetabolism in PreSxC9 in relation to healthy controls (pheight <0.001, at Cluster Level pFWE<0.05) Following Voxel-Based Partial Volume Corrections, Using SUVr Images eTable 1. Significant Areas of Decreased Cortical Glucose Metabolism in PreSxC9 Compared to HC, Identified Using Voxel-Wise Testing eTable 2. Significant Areas of Volume Decline in PreSxC9 Compared to HC, Identified Using Voxel-Wise Testing eTable 3. Significant Areas of Decreased Metabolism in PreSxC9 Compared to HC, Identified Using Voxel-Wise Testing Following Partial Volume Correction eTable 4. Significant Areas of Increased Cortical Glucose Metabolism in PreSxC9 Compared to HC, Identified Using Voxel-Wise Testing Following Partial Volume Correction eTable 5. A Volume-of-Interest (VOI)-Based Analysis After Region-Based Voxel-Wise (RBV) Correction for GM Atrophy, Using the Hammers N30R83 Maximum Probability Atlas eTable 6. Significant Areas of Increased Cortical Glucose Metabolism in PreSxC9 Compared to HC, Identified Using Voxel-Wise Testing Following Partial Volume Correction
