# Supplementary material for: Use of Multimodal Imaging and Clinical Biomarkers in Presymptomatic Carriers of C9orf72 Repeat Expansion
Source: JAMA Neurol. 2020 May 18;77(8):1–10. doi: 10.1001/jamaneurol.2020.1087 (PMC7417970; doi:10.1001/jamaneurol.2020.1087)
Supplement: Supplement. — eResults. Supplementary Analysis eReferences eFigure 1. Violin Plots of 18F FDG Uptake in Volumes of Interest, After Correcting for GM Atrophy With PVC, in Healthy Controls and PreSxC9 eFigure 2. W-Score Frequency Maps Detailing the Fraction of PreSxC9 With Suprathreshold W-Scores at Voxel Level eFigure 3. Mean W-Score Images Detailing Tracer Uptake in PreSxC9, Thresholded From 0.5 Standard Deviations From the Norm eFigure 4. Relative Hypermetabolism in PreSxC9 in relation to healthy controls (pheight <0.001, at Cluster Level pFWE<0.05) Following Voxel-Based Partial Volume Corrections, Using SUVr Images eTable 1. Significant Areas of Decreased Cortical Glucose Metabolism in PreSxC9 Compared to HC, Identified Using Voxel-Wise Testing eTable 2. Significant Areas of Volume Decline in PreSxC9 Compared to HC, Identified Using Voxel-Wise Testing eTable 3. Significant Areas of Decreased Metabolism in PreSxC9 Compared to HC, Identified Using Voxel-Wise Testing Following Partial Volume Correction eTable 4. Significant Areas of Increased Cortical Glucose Metabolism in PreSxC9 Compared to HC, Identified Using Voxel-Wise Testing Following Partial Volume Correction eTable 5. A Volume-of-Interest (VOI)-Based Analysis After Region-Based Voxel-Wise (RBV) Correction for GM Atrophy, Using the Hammers N30R83 Maximum Probability Atlas eTable 6. Significant Areas of Increased Cortical Glucose Metabolism in PreSxC9 Compared to HC, Identified Using Voxel-Wise Testing Following Partial Volume Correction [file jamaneurol-77-1008-s001.pdf]

Supplementary Online Content

De Vocht J, Blommaert J, Devrome M, et al. Use of multimodal imaging and clinical biomarkers in presymptomatic carriers of *C9orf72* repeat expansion. *JAMA Neurol.* Published online May 18, 2020. doi:10.1001/jamaneurol.2020.1087

eResults. Supplementary Analysis

eReferences

eFigure 1. Violin Plots of ^{18}F FDG Uptake in Volumes of Interest, After Correcting for GM Atrophy With PVC, in Healthy Controls and PreSxC9

eFigure 2. W-Score Frequency Maps Detailing the Fraction of PreSxC9 With Suprathreshold W-Scores at Voxel Level

eFigure 3. Mean W-Score Images Detailing Tracer Uptake in PreSxC9, Thresholded From 0.5 Standard Deviations From the Norm

eFigure 4. Relative Hypermetabolism in PreSxC9 in relation to healthy controls (pheight < 0.001, at Cluster Level pFWE< 0.05) Following Voxel-Based Partial Volume Corrections, Using SUVr Images

eTable 1. Significant Areas of Decreased Glucose Metabolism in PreSxC9 Compared to HC, Identified Using Voxel-Wise Testing

eTable 2. Significant Areas of Volume Decline in PreSxC9 Compared to HC, Identified Using Voxel-Wise Testing

eTable 3. Significant Areas of Decreased Glucose Metabolism in PreSxC9 Compared to HC, Identified Using Voxel-Wise Testing Following Partial Volume Correction

eTable 4. Significant Areas of Increased Cortical Glucose Metabolism in PreSxC9 Compared to HC, Identified Using Voxel-Wise Testing Following Partial Volume Correction

eTable 5. A Volume-of-Interest (VOI)-Based Analysis After Region-Based Voxel-Wise (RBV) Correction for GM Atrophy, Using the Hammers N30R83 Maximum Probability Atlas

eTable 6. Significant Areas of Increased Cortical Glucose Metabolism in PreSxC9 Compared to HC, Identified Using Voxel-Wise Testing Following Partial Volume Correction

This supplementary material has been provided by the authors to give readers additional information about their work.

eResults. Supplementary analysis

To address the concern that the observed clusters of relative hypermetabolism in PreSxC9 may be induced by global changes (reduced mean of that group), we examined relative hypermetabolism in PreSxC9 relation to healthy controls, by using the ratio over the FDG-PET uptake in the cerebellum, excluding structures reportedly affected by *C9orf72* mutation status, predominantly situated in the superior posterior lobe of the cerebellum and vermis in *C9orf72* mutation carriers.[1-4]

We therefor repeated the analysis using SUVR images corrected for the average uptake in cerebellar structures reportedly unaffected by carrying a C9orf72 mutation using the automated anatomical labelling atlas (lobule 3-5, 8 and 10), implemented in PMOD.

We present this data in the following figure (*eFigure 4*), which should be compared to *Figure 1D* in the main text, and table (*eTable 6*), which should be compared to *eTable 4*. From this analysis we found that the major conclusions were not altered by the different method of analysis.

eReferences

1. Bocchetta M, Cardoso MJ, Cash DM, Ourselin S, Warren JD, Rohrer JD. Patterns of regional cerebellar atrophy in genetic frontotemporal dementia. Neuroimage Clin. 2016;11:287-290.
2. Irwin DJ, McMillan CT, Brettschneider J, et al. Cognitive decline and reduced survival in *C9orf72* expansion frontotemporal degeneration and amyotrophic lateral sclerosis. J Neurol Neurosurg Psychiatry. 2013;84(2):163-169.
3. Mahoney CJ, Beck J, Rohrer JD, et al. Frontotemporal dementia with the C9ORF72 hexanucleotide repeat expansion: clinical, neuroanatomical and neuropathological features. Brain. 2012;135(pt 3):736-750.
4. Whitwell JL, Weigand SD, Boeve BF, et al. Neuroimaging signatures of frontotemporal dementia genetics: *C9ORF72*, tau, progranulin and sporadics. Brain. 2012;135(pt 3):794-806.

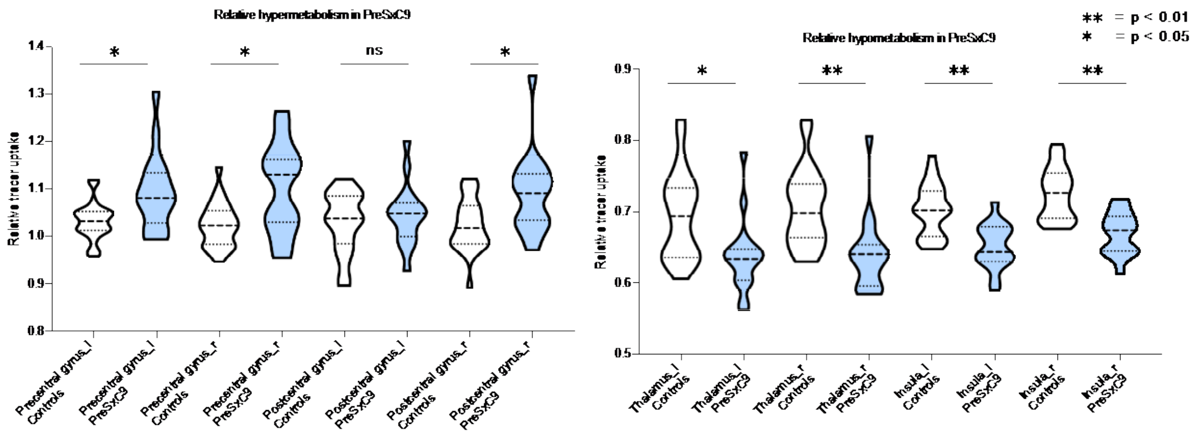

eFigure 1: Violin plots of ^{18}F FDG uptake in volumes of interest, after correcting for GM atrophy with PVC, in healthy controls (in white) and PreSxC9 (in blue). *This revealed significant differences in left and right thalamus, insular cortices, the precentral gyrus and right postcentral gyrus.*

eFigure 2: W-score frequency maps detailing the fraction of PreSxC9 with suprathreshold W-scores at voxel level. Section numbers refer to MNI coordinates.
A. represents the fraction of PreSxC9 with W-scores ≤ -1.96, drafted from FDG-PET images uncorrected for PVC. B. reveals the fraction of PreSxC9 with W-scores ≥ 1.96, drafted from FDG-PET images following PVC. C. reveals the fraction of PreSxC9 with W-scores ≤ -1.96, drafted from modulated GM images. Variation in colour depicts the fraction of PreSxC9 with significantly altered metabolism or modulated GM volume exceeding the threshold for abnormality.

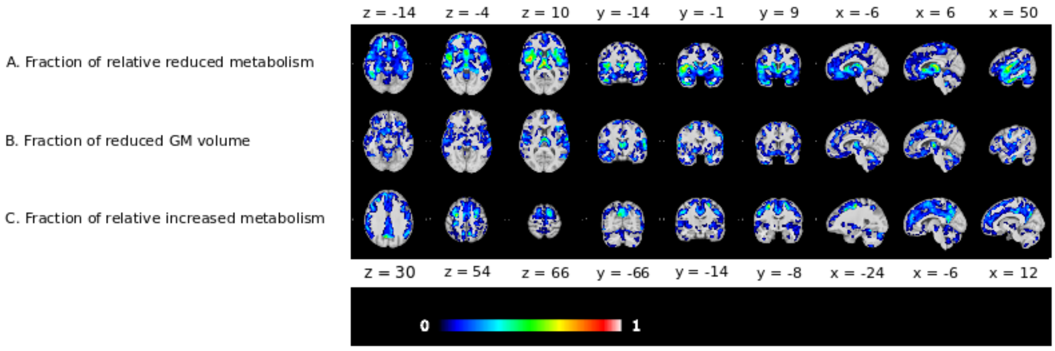

eFigure 3: Mean W-score images detailing tracer uptake in PreSxC9, thresholded from 0.5 standard deviations from the norm. Section numbers refer to MNI coordinates.
A. Mean W-score image detailing reduced metabolism in PreSxC9 without partial volume correction, B. Mean W-score image detailing increased metabolism in the PreSxC9 following partial volume correction.

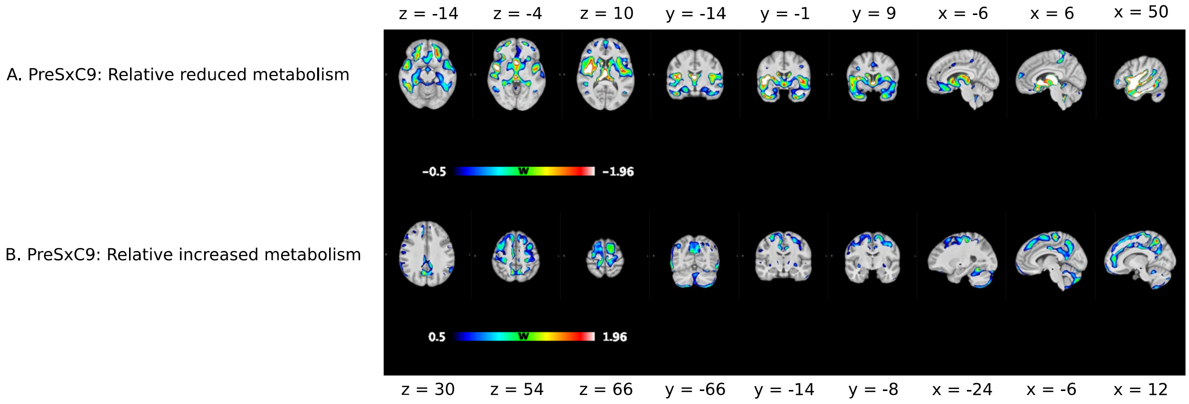

eFigure 4: Relative hypermetabolism in preSxC9 in relation to healthy controls (pheight < 0.001, at cluster level pFWE < 0.05) following voxel-based partial volume corrections, using SUVr images. Slice numbers refer to MNI coordinates.

FDG-PET PVC relative hypermetabolism

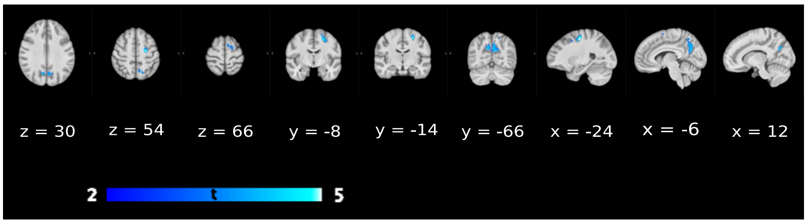

Cluster-level		peak-level		Peak voxel MNI coordinate			Cluster location	Maximum % change
$p_{FWE-corr}$	kE	$p_{FWE-corr}$	T	x	y	z	Anatomical Region	
0.001	8619	0.001	10.05	14	-34	6	Thalamus (pulvinar)	42%
				-14	-36	6	Thalamus (pulvinar)	
				4	-20	10	Thalamus (medial dorsal)	
				-26	6	-36	Temporal pole (BA 36)	
				4	6	-2	Caudate nucleus	
				8	0	14	Caudate nucleus	
				28	6	-38	Temporal pole (BA 36)	
				22	-16	-16	Parahippocampal gyrus	
				50	-8	10	Central opercular cortex (BA 48)	
				40	-12	16	Insular cortex (BA 13)	
				8	10	10	Caudate nucleus	
				48	-8	-32	Anterior inferior temporal gyrus (BA 20)	
				44	-44	-10	Inferior temporal gyrus (BA 37)	
				50	12	-14	Temporal pole (BA 38)	
				50	-24	-24	Inferior temporal gyrus (BA 20)	
				34	-40	0	n/a	
0.056	332	0.158	4.73	-22	28	-16	Orbitofrontal cortex (BA 11)	30%
				-22	44	-12	Orbitofrontal cortex (BA 11)	
				-12	34	-8	Orbitofrontal cortex (BA 11)	
0.035	388	0.320	4.41	24	28	-14	Orbitofrontal cortex (BA 11)	30%
				34	34	-10	Frontal pole (BA 47)	
				24	46	-12	Frontal pole (BA 11)	
				8	20	-18	Subcallosal cortex (BA 11)	

eTable 1: Significant areas of decreased cortical glucose metabolism in *preSxC9* compared to HC, identified using voxel-wise testing. Table shows all local maxima more than 1 cm apart.

Cluster-level		peak-level		Peak voxel MNI coordinate			Cluster location	Maximum % change
$p_{FWE-corr}$	kE	$p_{FWE-corr}$	T	x	y	z	Anatomical Region	
0.001	3951	0.006	6.23	-64	-9	7	Central opercular cortex (BA 22)	30%
				-43	-5	1	Insular cortex (BA 13)	
				-44	10	-5	Insular cortex (BA 13)	
				-67	-26	19	Anterior Supramarginal gyrus (BA 22)	
0.001	8522	0.016	5.89	7	-15	11	Thalamus (medial dorsal)	13%
				-4	-14	11	Thalamus (medial dorsal)	
				12	-26	5	Thalamus (pulvinar)	
				-12	-30	6	Thalamus (pulvinar)	

eTable 2: Significant areas of volume decline in *preSxC9* compared to HC, identified using voxel-wise testing. Table shows all local maxima more than 1 cm apart.

Cluster-level		peak-level		Peak voxel MNI coordinate			Cluster location	Maximum % change
$p_{FWE-corr}$	kE	$p_{FWE-corr}$	T	x	y	z	Anatomical Region	
0.001	2925	0.001	8.40	14	-32	4	Thalamus (pulvinar)	25%
				-12	-34	4	Thalamus (medial dorsal)	
				4	-18	10	Thalamus (medial dorsal)	
				22	-8	-16	Amygdala	
				-22	4	-34	Temporal pole (BA 28)	
				44	-4	-4	Insular cortex (BA 13)	
				54	10	-2	Temporal pole (BA 22)	
				40	4	-12	Insular cortex (BA 13)	
				-4	2	-8	n/a	
				4	-6	6	Thalamus	
				-16	-8	-20	Anterior Parahippocampal gyrus (BA 34)	
				4	-6	-10	Hypothalamus	
				26	4	-36	Temporal pole	
				-6	10	0	Caudate nucleus	
				32	-38	-6	Posterior Parahippocampal gyrus	
				8	14	0	Caudate nucleus	
0.010	281	0.026	5.55	52	-4	-34	Inferior temporal gyrus (BA 20)	18%
				44	16	-34	Temporal pole (BA 38)	
				52	10	-28	Temporal pole (BA 21)	

eTable 3: Significant areas of decreased metabolism in preSxC9 compared to HC, identified using voxel-wise testing following partial volume correction. Table shows all local maxima more than 1 cm apart.

Cluster-level		peak-level		Peak voxel MNI coordinate			Cluster location	Maximum % change
$p_{FWE-corr}$	kE	$p_{FWE-corr}$	T	x	y	z	Anatomical Region	
0.003	355	0.002	6.41	12	-68	28	Precuneous cortex	6%
				-4	-68	22	Precuneous cortex	
				-16	-64	56	Precuneous cortex (BA 7)	
				-14	-68	26	Precuneous cortex	
				-6	-60	54	Precuneous cortex	
				6	-62	18	Precuneous cortex	
				-6	-60	12	Posterior cingulate	
0.034	204	0.072	5.15	-24	-14	58	Precentral gyrus (BA 6)	5%
				-8	0	66	Supplementary motor cortex (BA 6)	
				-32	-6	50	Precentral gyrus (BA 6)	

eTable 4: Significant areas of increased cortical glucose metabolism in preSxC9 compared to HC, identified using voxel-wise testing following partial volume correction. Table shows all local maxima more than 1 cm apart.

Anatomical Region hammers (n30R83)	*Test statistic (Mann-Whitney U)*	p_{uncorr}*-value*	p_{corr}*-value (Benjamini-Hochberg)*
FL_mid_fr_G_l	202	0.79	0.83
FL_mid_fr_G_r	185	0.48	0.59
FL_precen_G_l	107	*0.007*	*0.02*
FL_precen_G_r	109	*0.008*	*0.03*
FL_strai_G_l	117	*0.01*	*0.04*
FL_strai_G_r	103	*0.005*	*0.02*
FL_OFC_AOG_l	121	*0.02*	*0.04*
FL_OFC_AOG_r	125	0.03	0.06
FL_inf_fr_G_l	187	0.51	0.61
FL_inf_fr_G_r	189	0.55	0.64
FL_sup_fr_G_l	183	0.45	0.56
FL_sup_fr_G_r	169	0.27	0.36
FL_OFC_MOG_l	109	*0.008*	*0.03*
FL_OFC_MOG_r	129	0.03	0.06
FL_OFC_LOG_l	154	0.13	0.21
FL_OFC_LOG_r	198	0.71	0.78
FL_OFC_POG_l	65	*0.0002*	*0.01*
FL_OFC_POG_r	120	*0.02*	*0.04*
Subgen_antCing_l	80	*0.001*	*0.01*
Subgen_antCing_r	161	0.19	0.28
Subcall_area_l	212	0.99	1
Subcall_area_r	102	*0.005*	*0.02*
Presubgen_antCing_l	142	0.07	0.12
Presubgen_antCing_r	168	0.25	0.35
Hippocampus_r	55	*0.00005*	*0.01*
Hippocampus_l	66	*0.0002*	*0.01*
Amygdala_r	122	*0.02*	*0.04*
Amygdala_l	133	0.04	0.08
Ant_TL_med_r	70	*0.0003*	*0.01*
Ant_TL_med_l	77	*0.001*	*0.01*
Ant_TL_inf_lat_r	140	0.06	0.11
Ant_TL_inf_lat_l	129	0.03	0.06
G_paraH_amb_r	92	*0.002*	*0.01*
G_paraH_amb_l	150	0.11	0.18
G_sup_temp_post_r	212	0.99	1
G_sup_temp_post_l	182	0.43	0.55
G_tem_midin_r	102	*0.005*	*0.02*
G_tem_midin_l	68	*0.0002*	*0.01*
G_fus_r	106	*0.006*	*0.02*
G_fus_l	95	*0.003*	*0.02*
Post_TL_l	113	*0.01*	*0.03*
Post_TL_r	103	*0.005*	*0.02*
G_sup_temp_ant_l	100	*0.004*	*0.02*
G_sup_temp_ant_r	57	*0.00007*	*0.01*
PL_postce_G_l	199	0.73	0.78
PL_postce_G_r	105	*0.006*	*0.02*
PL_sup_pa_G_l	150	0.11	0.18
PL_sup_pa_G_r	193	0.62	0.7
PL_rest_l	166	0.23	0.33
PL_rest_r	194	0.64	0.71
Insula_l	66	*0.0002*	*0.01*
Insula_r	50	*0.00003*	*0.01*
G_cing_ant_l	189	0.55	0.64
G_cing_ant_r	206	0.87	0.91

G_cing_post_l	165	0.22	0.33
G_cing_post_r	170	0.28	0.37
Cerebellum_r	209	0.93	0.96
Cerebellum_l	177	0.36	0.47
Putamen_l	135	0.05	0.09
Putamen_r	113	*0.011*	*0.03*
Thalamus_l	98	*0.003*	*0.02*
Thalamus_r	66	*0.0002*	*0.01*
CaudateNucl_l	76	*0.0005*	*0.01*
CaudateNucl_r	99	*0.004*	*0.02*

eTable 5: A volume-of-interest (VOI)-based analysis after region-based voxel-wise (RBV) correction for GM atrophy, using the Hammers N30R83 Maximum Probability atlas.

Cluster-level		peak-level		Peak voxel MNI coordinate			Cluster location	Maximum % change
$p_{FWE-corr}$	kE	$p_{FWE-corr}$	T	x	y	z	Anatomical Region	
0.054	293	0.032	5.26	-24	-12	52	Precentral gyrus (BA 6)	4%
				-8	2	66	Superior frontal gyrus (BA 6)	
				-30	0	52	Middle frontal gyrus (BA 6)	
				-18	-4	66	Superior frontal gyrus (BA 6)	
				-24	8	48	Superior frontal gyrus	
0.010	487	0.080	4.90	12	-68	28	Precuneous cortex	5%
				-16	-62	56	Precuneous cortex (BA 7)	
				-6	-68	24	Precuneous cortex	
				-6	-66	34	Precuneous cortex	
				8	-62	20	Precuneous cortex	

eTable 6: Significant areas of increased cortical glucose metabolism in preSxC9 compared to HC, identified using voxel-wise testing following partial volume correction. Table shows all local maxima more than 1 cm apart.
